# Decrease in Shiga toxin expression using a minimal inhibitory concentration of rifampicin followed by bactericidal gentamicin treatment enhances survival of *Escherichia coli *O157:H7-infected BALB/c mice

**DOI:** 10.1186/1476-0711-10-34

**Published:** 2011-09-12

**Authors:** Elias A Rahal, Natalie Kazzi, Ahmad Sabra, Alexander M Abdelnoor, Ghassan M Matar

**Affiliations:** 1 Department of Microbiology and Immunology, Faculty of Medicine, American University of Beirut, Beirut, Lebanon

**Keywords:** *Escherichia coli *O157:H7, rifampicin, gentamicin, Shiga toxins

## Abstract

**Background:**

Treatment of *Escherichia coli *O157:H7 infections with antimicrobial agents is controversial due to an association with potentially fatal sequelae. The production of Shiga toxins is believed to be central to the pathogenesis of this organism. Therefore, decreasing the expression of these toxins prior to bacterial eradication may provide a safer course of therapy.

**Methods:**

The utility of decreasing Shiga toxin gene expression in *E. coli *O157:H7 with rifampicin prior to bacterial eradication with gentamicin was evaluated *in vitro *using real-time reverse-transcription polymerase chain reaction. Toxin release from treated bacterial cells was assayed for with reverse passive latex agglutination. The effect of this treatment on the survival of *E. coli *O157:H7-infected BALB/c mice was also monitored.

**Results:**

Transcription of Shiga toxin-encoding genes was considerably decreased as an effect of treating *E. coli *O157:H7 *in vitro *with the minimum inhibitory concentration (MIC) of rifampicin followed by the minimum bactericidal concentration (MBC) of gentamicin (> 99% decrease) compared to treatment with gentamicin alone (50-75% decrease). The release of Shiga toxins from *E. coli *O157:H7 incubated with the MIC of rifampicin followed by addition of the MBC of gentamicin was decreased as well. On the other hand, the highest survival rate in BALB/c mice infected with *E. coli *O157:H7 was observed in those treated with the *in vivo *MIC equivalent dose of rifampicin followed by the *in vivo *MBC equivalent dose of gentamicin compared to mice treated with gentamicin or rifampicin alone.

**Conclusions:**

The use of non-lethal expression-inhibitory doses of antimicrobial agents prior to bactericidal ones in treating *E. coli *O157:H7 infection is effective and may be potentially useful in human infections with this agent in addition to other Shiga toxin producing *E. coli *strains.

## Background

*Escherichia coli *O157:H7 is the most commonly encountered member of the Enterohemorrhagic *Escherichia coli *(EHEC) group. Infection with this agent typically results in bloody diarrhea with low-grade or absence of fever with no leukocytes in the stools [1]. Symptoms may progress, culminating in potentially fatal complications such as the hemolytic uremic syndrome (HUS) [2-4] and thrombotic thrombocytopenia purpura (TTP) in the elderly and the young [3]. This organism causes about 73,000 illnesses annually in the United States [5].

Until recently, the most common mode of *E. coli *O157:H7 infection was via the oral route by consumption of ground beef. In recent years, *E. coli *O157:H7 has been isolated with increasing frequency from fresh produce. Other modes of infection include consumption of animal products, person-to-person transmission, waterborne, animal contact and less commonly, laboratory-associated transmission [6]. Several virulence factors contribute to the pathogenicty of *E. coli *O157:H7 with the production of Shiga toxins (Stxs) being at the epicenter of the infectious process. These toxins consist of two major groups: Stx1, which is nearly identical to the toxin of *Shigella dysenteriae *type 1, and Stx2, which shares less than 55% amino acid sequence with Stx1 [7-9]. Another virulence factor is the locus of enterocyte effacement (LEE) which contains genes required for the production of the attaching and effacing (A/E) lesions that accompany *E. coli *O157: H7 infection [10]. These genes basically allow the bacteria to colonize the intestines. The Shiga toxins have also been implicated in contributing to the process of intestinal colonization [11].

Treating *E. coli *O157:H7 infection with antimicrobial agents is currently contraindicated due to its association with HUS and an increased case-fatality rate [12-14]. This may be due to the activation of a stress response signal upon treatment with the antimicrobial agent that potentially leads to enhanced production and subsequent release of Shiga toxins [15]. Alternatively, the antimicrobial agent may lead to bacterial lysis and subsequent release of stored toxins that are present in the periplasmic space [16].

A potential method of treatment may involve administration of an antimicrobial agent at non-bactericidal doses that limit toxin expression prior to employing an agent at bactericidal doses. This would decrease toxin production prior to bacterial cell lysis and hence may circumvent the sequelae associated with this type of infection. We have previously demonstrated that minimum inhibitory concentrations (MIC) of rifampicin effectively decreased toxin release from *E. coli *O157:H7 *in vitro *[17,18]. We have also shown that this agent improves the survival rate of mice infected with *E. coli *O157:H7 [19]. Employment of rifampicin in monotherapy is associated with evolution of rapid resistance [20]. Hence, we investigated the utility treating infected mice with rifampicin at doses that limit toxin expression followed by gentamicin at bactericidal doses to eradicate the bacterial agent.

## Methods

### *In vitro *antimicrobial susceptibility testing

The minimum inhibitory concentration (MIC) and minimum bactericidal concentration (MBC) of rifampicin and gentamicin for the CDC 26-98 strain *E. coli *O157:H7 were determined as previously described [19].

### Real-time reverse-transcription polymerase chain reaction (RT-PCR) for assessing toxin gene transcription

To assess the effect of treating *E. coli *O157:H7 cells with rifampicin and gentamicin on toxin gene expression multiple regimens were tested. An inoculum of 10^6 ^CFU of the CDC 26-98 strain of *E. coli *O157:H7 in 2 ml of Mueller Hinton broth was incubated in the MIC of rifampicin for 18 hrs at 37°C. A similar inoculum was incubated in the MBC of gentamicin for 18 hrs at 37°C. A different sample was incubated in the MIC of rifampicin for 18 hrs at 37°C, cells were then centrifuged (5000 rpm, 5 min), and resuspended in the MBC of gentamicin prior to incubation for 4 hrs at 37°C. Similarly a sample was incubated in the MIC of rifampicin for 18 hrs at 37°C. Cells were then centrifuged and resuspended in the MBC of rifampicin prior to further incubation for 4 hrs at 37°C. An inoculum of *E. coli *O157:H7 grown in 2 ml of antimicrobial agent-free broth for 22 hrs at 37°C was also included as a normal growth control. None of the samples incubated with the antimicrobial agents showed any growth.

After incubations, total RNA was extracted from 10^6 ^CFU of each of the samples described above using the Illustra RNAspin Mini RNA Isolation Kit (General Electric Company, United Kingdom) according to the manufacturer's specifications for bacterial cells. Reverse transcription and cDNA synthesis was then performed on all samples of extracted RNA using the QuantiTect^® ^Reverse Transcription Kit (QIAGEN, Germany) according to the manufacturer's instructions. Gene expression was then assessed with real-time PCR for the *stx1 *and *stx2 *genes, that respectively encode Shiga toxin 1 (Stx1) and Shiga toxin 2 (Stx2). This was performed using a BioRad CFX96 Real Time System, C1000 Thermal Cycler (Germany). Primers were obtained from Thermo Scientific (Ulm, Germany). Previously published primers were used for detection of *stx1 *and *stx2 *transcripts [21]. Reactions (20 μl), performed in triplicates per sample, each contained 738 ng cDNA, 10 pmoles of each primer and 1 × QuantiFast SYBR green PCR mix (Qiagen, germany). Reactions were incubated at 95°C for 15 minutes followed by 45 cycles of 95°C for 10 seconds, 55°C for 10 seconds and 72°C for 20 seconds.

Relative expression (RE) was calculated using the formula: RE = (1+ % E) ^Δ**Ct**^, where E is the efficiency of the real-time run and ΔCt is the difference between the Ct value of samples extracted from *E. coli *O157: H7 grown in the absence of drugs and the Ct value of the antimicrobial agent-treated samples. In our experiments, we used an efficiency value of 100%, therefore, the equation employed for the analysis was: RE = (1 + 1) ^Δ**Ct **^= (2) ^Δ**Ct**^. Expression levels were normalized to those detected in bacterial samples incubated with drug-free media.

### Reverse passive latex agglutination (RPLA) for assessing toxin release

VTEC-RPLA "SEIKEN" kit (Denka Seiken, LTD., Tokyo, Japan) was used to assess Stx1 and Stx2 release from the CDC 26-98 strain of *E. coli *O157:H7 incubated in the presence of the MIC of rifampicin followed by the MBC of gentamicin. *E. coli *O157:H7 was grown for 18 hrs at 37°C in rifampcin-containing TSB followed by additional 4 hrs of incubation with the MBC of gentamicin. Toxin titers were then determined by reverse passive latex agglutination (RPLA) and compared to *E. coli *O157:H7 grown in antimicrobial free TSB. When toxin titers were tested in bacterial cultures grown in the MIC or MBC of rifampicin, growth inhibition was accounted for and CFU numbers were adjusted to be equivalent to those grown in antimicrobial agent-free media.

### Antimicrobial treatment of *E. coli *O157:H7-infected BALB/c mice

Adult male BALB/c mice, 4-6 weeks old and weighing 22-39 g each, were obtained from the Animal Care Facility at the American University of Beirut (AUB). The LD50 of the CDC 26-98 strain of *E. coli *O157:H7 in these mice was determined as previously described [19]. To assess the utility of an antimicrobial regimen for the treatment of infected mice, seven groups each containing 8 mice were used (Table 1). Mice received 3 × LD50 of the CDC 26-98 strain of *E. coli *O157:H7 and then were treated with the *in vivo *MIC equivalent dose of of rifampicin (15.168 μg), the *in vivo *MBC equivalent dose of gentamicin (7.584 μg) or with both successively. A group that was treated with the *in vivo *MIC equivalent dose of rifampicin followed by the MBC equivalent dose of the same agent was also assessed. Groups of mice that were not infected but injected with sterile broth or with the antimicrobial agents in addition to a group that was infected but not treated were included as controls. All injections were administered intraperitoneally and their volumes did not exceed 0.5 ml/mouse/day. Mice were then monitored for death and weight change over a period of 14 days. Mice were to be euthanized had they lost more than 30% of their body weight post-infection; however, this did not occur during the 14 day monitoring period.

**Table 1:** Mouse treatment regimen

Group	First Injection (Hour 0)	Second Injection (Hour 1)	Third Injection (Hour 17)
**1**	*E. coli *O157:H7 (3 × LD50)	Rifampicin (MIC)	-
**2**	*E. coli *O157:H7 (3 × LD50)	Gentamicin (MBC)	-
**3**	*E. coli *O157:H7 (3 × LD50)	Rifampicin (MIC)	Gentamicin (MBC)
**4**	*E. coli *O157:H7 (3 × LD50)	Rifampicin (MIC)	Rifampicin (MBC)
**5**	Trypticase soy broth	Trypticase soy broth	Trypticase soy broth
**6**	Trypticase soy broth	Rifampicin (MIC)	Gentamicin (MBC)
**7**	*E. coli O157:H7 *(3 × LD50)	-	-

The therapeutically relevant *in vivo *MIC equivalent dose of rifampicin was extrapolated from its *in vitro *MIC according to the following formula: rifampicin *in vivo *MIC dose (μg) = [rifampicin *in vitro *MIC (μg/μl) × *in vitro *MIC broth volume (μl) × *E. coli *O157:H7 CFU administered *in vivo*]/*E. coli *O157:H7 CFU per *in vitro *MIC reaction. Consequently, the ratio of rifampicin to *E. coli *O157:H7 CFU determined by *in vitro *MIC testing was maintained *in vivo*. Similarly, the therapeutically relevant *in vivo *MBC equivalent dose of rifampicin was extrapolated from its *in vitro *MBC according to the following formula: rifampicin *in vivo *MBC dose (μg) = [rifampicin *in vitro *MBC (μg/μl) × *in vitro *MBC broth volume (μl) × *E. coli *O157:H7 CFU administered *in vivo*]/*E. coli *O157:H7 CFU per *in vitro *MBC reaction. The same formula was used to determine the *in vivo *MBC equivalent dose of gentamicin.

## Results

### Effect of *in vitro *treatment with antimicrobial agents on toxin expression in *E. coli *O157:H7

**Figure 1:**
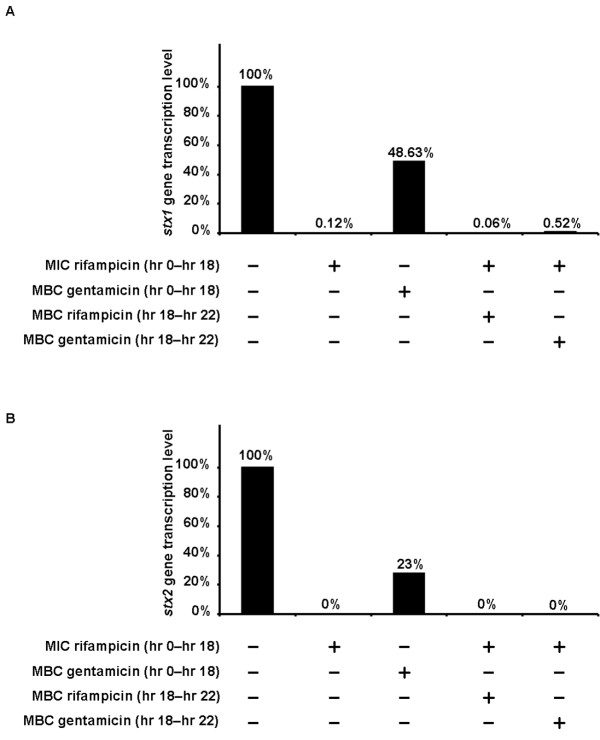
**Relative transcription levels of the *stx1 *and *stx2 *genes in *E. coli *O157:H7 treated with antimicrobial agents**. Bacterial inocula were either grown in antimicrobial-agent free broth, treated with the minimal inhibitory concentration (MIC) of rifampicin or with the minimal bactericidal concentration (MBC) of gentamicin. One sample was treated with the MIC of rifampicin followed by the MBC of rifampicin itself while another was treated with the MIC of rifampicin followed by the MBC of gentamicin. RNA was then extracted from these samples. Subsequently, the relative transcription levels of the *stx1 *and *stx2 *genes were assessed with real-time RT-PCR as described in the materials and methods section. All expression levels were normalized to those detected in bacteria grown in antimicrobial agent-free broth. (A) Relative transcription levels of the *stx1 *gene. (B) Relative transcription levels of the *stx2 *gene.

Multiple rifampicin and gentamicin treatment regimens were used to assess the effect of these agents on the expression of Stx1 and Stx2 encoding genes, *stx1 *and *stx2*, in *E. coli *O157:H7. Treatments tested are described in the materials and methods section. Real-time RT-PCR showed that the *stx1 *and *stx2 *genes were expressed in the *E. coli *O157:H7 strain employed when incubated in antimicrobial agent-free broth (Figure 1). After incubation with antimicrobial agents, levels of *stx1 *gene expression markedly decreased (> 99% decrease) in the sample treated with the MIC of rifampicin (8 μg/ml). A similar decrease was observed in the sample treated with the MIC of rifampicin followed by the MBC of rifampicin (16 μg/ml) and in the sample treated with the MIC of rifampicin followed by the MBC of gentamicin (4 μg/ml). The least inhibition of toxin gene expression (51.37% decrease) was seen in the sample treated with the MBC of gentamicin. A marked decrease in *stx2 *transcript detection was observed in the sample treated with the MBC of gentamcin (77% decrease.) On the other hand, *stx2 *expression was completely inhibited by treatment with the MIC of rifmapicin, treatment with the MIC of rifampicin followed by the MBC of rifampicin, and treatment with the MIC of rifampicin followed by the MBC of gentamicin.

Treatment of *E. coli *O157:H7 with the MIC of rifampicin followed by the MBC of gentamicin showed an 8-fold decrease of Stx1 toxin release into the growth medium. On the other hand, there was no change in the level of Stx2 released into the growth medium as compared to toxin titers of *E. coli *O157:H7 grown in antimicrobial free broth (data not shown.)

### Effect of antimicobial treatment on *E. coli *O157:H7-infected mice

We have previously shown that treatment with the MIC of rifamipicin was capable of significantly decreasing the expression and release of both Stx1 and Stx2 from *E. coli *O157:H7 [17-19]. This may be employed in a treatment regimen whereby toxin production is limited prior to administering an antimicrobial agent that can effectively kill the bacteria. Therefore, we assessed this treatment approach *in vivo*.

**Figure 2:**
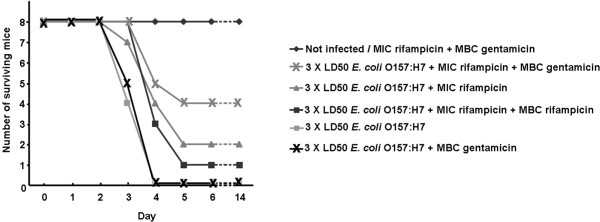
**Number of surviving BALB/c mice after infection with *E. coli *O157:H7 and treatment with antimicrobial agents**. Male BALB/c mice were infected with *E. coli *O157:H7 and then treated with rifampicin and/or gentamicin as delineated in the materials and methods section. Mice were then monitored for 14 days.

Mice were infected with 3 × LD50 of *E. coli *O157:H7 (equivalent to 9.48 × 10^5 ^CFU) and treated with various regimens of rifampicin and gentamicin as summarized in Table 1 and described in the materials and methods section. All mice infected with *E. coli *O157:H7 and left untreated or treated with the *in vivo *MBC equivalent dose of gentamicin were dead by day 4 post infection (Figure 2). Mice in other groups that survived until day 5 remained alive for the rest of the 14 day monitoring period. The highest survival rate was obtained with the group treated with the *in vivo *MIC equivalent dose of rifampicin followed by the *in vivo *MBC equivalent dose of gentamicin. In this group, 50% of the mice infected and treated were alive on day 5 and remained so forward. In comparison, 25% of the mice infected and treated with the *in vivo *MIC equivalent dose of rifampicin were alive on day 5. On the other hand, mice treated post-infection with the *in vivo *MIC equivalent dose of rifampicin followed by the *in vivo *MBC equivalent dose of rifampicin showed a 12.5% survival rate.

## Discussion

Using antimicrobial agents to treat *E. coli *O157:H7 infections has been contraindicated due to studies showing an association between antimicrobial treatment and increased fatality rates [4]. The quest for other treatments has led to the development of antibodies, among other agents, aimed at direct inhibition of the toxins secreted by *E. coli *O157:H7 and associated with the severe sequelae of infection [22,23]. While these approaches appear to be effective, their affordability limits their use. On the other hand, the use of probiotics, physical means in addition to natural and chemical products for the treatment and prevention of *E. coli *O157:H7 has been assessed by multiple groups with variable success [24-28].

Antimicrobial agents remain to be the method of choice for early empirical treatment of bacterial infections, particularly in the treatment of gastroenteritis in pediatric patients [26]. Antimicrobial treatment for *E. coli *O157:H7 may be possible if Shiga toxin expression can first be decreased before administering bactericidal doses of an agent, thus limiting potential toxin release upon lysis of the organism. This was previously established by our group *in vitro *[17,18] and by the study at hand *in vivo*.

Upon establishing that *in vitro *treatment of *E. coli *O157:H7 inocula with the MIC of rifampicin followed by the MBC of gentamicin potently decreases the transcription of the Stx1 and Stx2 encoding genes, we assessed the effect of this treatment mode on toxin release. Testing the levels of toxins released into the growth medium showed an 8-fold decrease in Stx2 levels whereas no such decrease was observed for Stx1 levels. This may be explained by the biology of production and storage of these toxins and their turnover rates. Stx1 is stored in the periplasmic space; consequently, upon addition of gentamicin at a bactericidal concentration, cells possibly ruptured and released the pre-stored Stx1. On the other hand, Stx2 is typically found in the extracellular fraction and is released from bacterial cells [29].

Treatment of infected mice with the *in vivo *MIC equivalent dose of rifampicin, followed by the *in vivo *MBC equivalent dose of gentamicin increased the survival rate of infected mice by 50%. On the other hand treatment with MBC equivalent dose of gentamicin led to the death of all mice that received this agent. Rifampicin, being a known inhibitor of gene transcription, is assumed here to have hindered the expression of the toxins by *E. coli *O157:H7. After suppression of toxin expression with rifampicin, treatment with gentamicin helped eradicate the infection and enhance mouse survival.

In a previous report [18], a higher fold-decrease in toxin-release was seen *in vitro *when *E. coli *O157:H7 was incubated with rifampicin or gentamicin alone compared to the decrease observed in the present study upon combinatorial treatment. However, treatment of infected mice with rifampicin followed by gentamicin proved to be more effective than when these antimicrobial agents were used individually. Therefore, *in vivo*, these antimicrobial agents may have had other effects additional to affecting toxin expression and release. These agents, for example, may have had a direct inhibitory effect on the bacterial lipopolysacchride consequently leading to decreased inflammation and septic shock [30].

In addition, the combinational treatment of rifampicin and gentamicin *in vivo *proved to be more effective than treating with rifampicin alone, that is, treating the infected mice with an MIC dose of rifampicin first, followed later on by a bactericidal dose of the same drug. A potential reason behind this is a resistance to rifampicin that may have developed, and thus resulted in the ineffective outcome of using an MBC dose of rifampicin, as compared to that of gentamicin. Resistance to rifampicin by *E. coli *was reported shortly after rifampicin was discovered. Susceptible bacteria, like *E. coli*, develop resistance to rifampicin by one-step mutations that alter the subunit structure of the RNA-polymerase, and this takes place rapidly when rifampicin is used alone [20,31].

## Conclusions

The present study indicates that treatment with rifampicin followed by gentamicin is capable of decreasing toxin expression in *E. coli *O157:H7 *in vitro *and improving the survival of mice infected with this organism. Further investigations should indicate cases where such a treatment modality would be of benefit. Antibacterial agents should therefore not be kept out of perspective for the treatment of *E. coli *O157:H7 among other Shiga toxin-producing organisms. However, care should be given to the selection of agents with the appropriate mechanism of action. The effect of these agents on toxin gene expression should be taken into account. Agents that limit toxin production prior to eradicating the bacterial infection are preferable. This may be achieved by combination antimicrobial therapy such as the one described herein.

## Competing interests

The authors declare that they have no competing interests.

## Authors' contributions

EAR and GMM conceived and designed the study, monitored the progress and supervised and drafted the manuscript. NK carried out the in vitro and in vivo assays described herein and participated in drafting the manuscript. AS participated in designing and performing the real-time reverse-transcription polymerase chain reaction assays. AMA participated in designing the in vivo monitoring aspects of this study and in manuscript revisions. All authors have read and approved the final manuscript.
